# Sleep patterns and their correlation with cardiovascular health in the general population: A cross-sectional study

**DOI:** 10.6026/9732063002001939

**Published:** 2024-12-31

**Authors:** Ramya Sampathkumar, Pradeepan Sengiah Ramaswamy, Aadhithya Raaj Pandurangan, Sushmitha Rameshbabu, Saranya R., Shanmukha Vinay

**Affiliations:** 1 Department of Internal Medicine, Blackpool Victoria Teaching Hospital, Blackpool, United Kingdom; 2 Department of Internal Medicine, Madras Medical College, Chennai, Tamil Nadu, India; 3 Department of Community Medicine, Madha Medical College and Research Institute, Chennai, Tamil Nadu, India; 4 Department of Cardiology, Care Hospitals, Visakhapatnam andhra Pradesh, India

**Keywords:** Sleep patterns, cardiovascular health, sleep duration, sleep quality, cardiovascular risk, framingham risk score

## Abstract

Sleep patterns, including duration and quality, are closely linked to cardiovascular health. This cross-sectional study of 100 participants aged 30-65 years assessed sleep patterns using validated questionnaires and measured cardiovascular health using the Framingham Risk Score. Short sleep duration (<6 hours) and poor sleep quality were significantly associated with higher cardiovascular risk (p < 0.001), while optimal sleep duration (7-8 hours) correlated with the lowest risk scores (p = 0.002). Long sleep duration also increased cardiovascular risk, particularly in individuals with conditions such as obesity and hypertension. These findings underscore the importance of promoting healthy sleep habits as a key strategy in preventing cardiovascular disease in the general population.

## Background:

Cardiovascular diseases (CVD) are still the most common cause of morbidity and mortality in the world. Classic risk factors include hypertension, hyperlipidaemia, smoking and obesity, but evidence has been mounting in recent years to suggest that sleep patterns, including sleep duration and quality, play a role in cardiovascular health [1, 2]. Both short sleep duration, less than 6 hours per night and long sleep duration, more than 9 hours per night, has been linked to increased risks of cardiovascular diseases, which include hypertension, coronary artery disease and stroke [3, 4]. Another is sleeping quality. Inferior quality is sleeping, with waking up at least once in a while at night, the patient may complain of not being able to fall asleep or restless sleep, associated with hypertension and risk of heart disease in higher rates than those with better sleep quality [5, 6]. Despite the burgeoning evidence already linking sleep and cardiovascular health, there are few studies conducted on the general population, focusing on sleep duration and quality and their impact on cardiovascular risk [7]. The current cross-sectional study is aimed at exploring the relationship of sleep patterns with cardiovascular health within the general population [8]. Therefore, it is of interest to assess sleep duration, the quality of sleep and various cardiovascular risk factors including hypertension, BMI and cholesterol levels such that it may be evaluated whether improvement in sleep habits can reduce the risk of cardiovascular disease [9, 10].

## Materials & Methods:

This was a cross-sectional study that was conducted between the months of January 2023 and December 2023. The participants were approached from the community centre and the health clinics. It was ensured that the selection was conducted in such a manner that diversity would be achieved among various age groups, genders and socioeconomic status. The inclusion and exclusion criteria are stated below.

## Inclusion criteria:

[1] Age range of between 30 to 65 years

[2] Participants without any known disease of the cardiovascular system.

## Exclusion criteria:

[1] Participants diagnosed with sleep disorders such as sleep apnea.

[2] Those who are on sleep medications.

## Study design:

Participants completed validated questionnaires assessing their sleep patterns, including sleep duration and sleep quality (using the Pittsburgh Sleep Quality Index or PSQI), as well as cardiovascular risk factors such as hypertension, cholesterol levels, BMI and smoking status.

## Sleep variables assessed:

[1] Sleep duration: Defined as short (<6 hours), optimal (7-8 hours) and long (>9 hours).

[2] Sleep quality: Evaluated using the PSQI, where >5 = poor sleep quality.

## Cardiovascular risk assessment:

Cardiovascular health was assessed by means of the Framingham Risk Score, whereby a patient's 10-year risk to develop cardiovascular disease is estimated on the basis of age, blood pressure, cholesterol level, smoking status and diabetes.

## Data collection:

[1] Sleep duration and quality measured by the individual participants through the use of PSQI.

[2] Cardiovascular Health Measured by Blood pressure collection, cholesterol levels collection, collection of the BMI as well as by the report from the individual participants on their smoking status and physical activities.

[3] Framingham Risk Score Calculated based on cardiovascular data collected.

## Statistical analysis:

The data were analyzed using SPSS software, version 26. Continuous variables are reported as mean ± standard deviation, while categorical variables are expressed as percentages. Comparisons of outcomes between distinct groups of sleep duration were done by applying chi-square and t-tests. A p-value < 0.05 was considered statistically significant.

## Results:

A total of 100 participants completed the study. The distribution of sleep duration, sleep quality and cardiovascular risk scores are presented in the following tables. Where in the age was 47.6 and there was roughly the same number of males as females. Hypertension prevalence was at 32% (Table 1). Distribution of Sleep Duration, that more people reported sleep durations close to optimal; however, short or long sleep was recorded in 20% (Table 2). Inferior quality sleep was reported by 32% of participants. It means that a substantial segment of the population reported problems with sleep (Table 3). The Framingham Risk Scores were significantly lower in the subjects who had ideal sleep time than in those with short or long sleep times (Table 4). Poor sleep quality is significantly associated with a higher prevalence of hypertension (Table 5). Short sleep duration subjects had a significantly higher BMI compared to ideal sleep duration subjects (Table 6). Smoking status was not significantly associated with sleep duration (Table 7). The physical activity of the participants was high if they slept well and vice versa (Table 8). The normal sleep duration has a large cholesterol level than short and long sleep duration (Table 9). The higher cardiovascular risk score was shown by the subjects who took a prolonged period to fall asleep (Table 10).

## Discussion:

This study demonstrates a significant correlation between sleep patterns and cardiovascular health in the general population [11]. Short sleep duration (<6 hours) and poor sleep quality were associated with higher cardiovascular risk, including elevated Framingham Risk Scores and a higher prevalence of hypertension [12, 13]. These findings are consistent with previous research linking sleep disruptions to adverse cardiovascular outcomes [14, 15]. The relation of optimally long sleep duration, that is, 7-8 hours, with better cardiovascular health was because it was related to lower Framingham Risk Scores, lower BMI and lower cholesterol levels. It may be assumed that having healthy sleep duration could significantly contribute to the reduction of cardiovascular risk [16]. Inversely, long sleep duration more than 9 hours was related to increased cardiovascular risk and this effect was significant only for patients who had comorbid conditions such as obesity and hypertension. This agrees with studies that suggest both extremes of sleep duration are detrimental to cardiovascular health [17]. Poor sleep quality was also associated with the presence of hypertension and a lower level of physical activity, two known contributors to risk for cardiovascular disease. Such factors are an important additional reason improving sleep quality may have the potential to play a significant role in reducing cardiovascular disease burden in the general population [18]. Melatonin can, via its scavenging and antioxidant nature, improve endothelial function via the increased availability of nitric oxide, thereby exerting vasodilatory and hypotensive effects, it can also interfere with the peripheral and central autonomic nervous systems, with a subsequent decrease in the tone of the adrenergic system and an increase in the cholinergic system [19].

## Conclusion:

The effects of sleep duration and quality on cardiovascular health are adequately documented. Shorter and more than optimal sleep durations pose an increased risk for cardiovascular illnesses, while optimal sleep of 7-8 hours is associated with better outcomes for cardiovascular health. It should also be considered the improvement in sleep quality with adequate sleep duration as crucial components of cardiovascular disease preventive strategies for the general populace.

## Figures and Tables

**Table 1:** Baseline characteristics of participants

**Characteristic**	**Value (n = 100)**
Age (Mean ± SD)	47.6 ± 9.4
Gender (Male)	52:48:00
Body Mass Index (BMI)	27.4 ± 3.7
Hypertension (%)	32%
Smoking Status (%)	Current: 18%

**Table 2:** Distribution of sleep duration

**Sleep Duration Category**	**Percentage of Participants (%)**
Short Sleep (<6 hours)	20%
Optimal Sleep (7-8 hours)	60%
Long Sleep (>9 hours)	20%

**Table 3:** Sleep quality (Pittsburgh sleep quality index)

**PSQI Score Category**	**Percentage of Participants (%)**
Good Sleep Quality (≤5)	68%
Poor Sleep Quality (>5)	32%

**Table 4:** Cardiovascular risk (Framingham risk score)

**Framingham Risk Score**	**Mean ± SD**	**p-value**
Short Sleep (<6 hours)	15.2 ± 3.6	
Optimal Sleep (7-8 hours)	10.8 ± 2.9	0.003
Long Sleep (>9 hours)	14.8 ± 3.9	

**Table 5:** Association between sleep quality and hypertension

**Sleep Quality**	**Hypertension Prevalence (%)**	**p-value**
Good Sleep Quality (≤5)	28%	
Poor Sleep Quality (>5)	42%	0.045

**Table 6:** Relationship between sleep duration and BMI

**Sleep Duration Category**	**Mean BMI (kg/m** ^2^ **)**	**p-value**
Short Sleep (<6 hours)	28.6 ± 4.1	
Optimal Sleep (7-8 hours)	26.4 ± 3.5	0.004
Long Sleep (>9 hours)	28.0 ± 3.9	

**Table 7:** Relation between smoking status and sleep duration

**Smoking Status**	**Short Sleep (%)**	**Optimal Sleep (%)**	**Long Sleep (%)**	**p-value**
Current Smokers	21%	15%	20%	0.245
Former Smokers	25%	22%	26%	
Never Smokers	54%	63%	54%	

**Table 8:** Relation between physical activity and sleep quality

**Sleep Quality**	**High Physical Activity (%)**	**Low Physical Activity (%)**	**p-value**
Good Sleep Quality (≤5)	65%	35%	
Poor Sleep Quality (>5)	40%	60%	0.032

**Table 9:** Association between sleep duration and cholesterol levels

**Sleep Duration Category**	**Mean Cholesterol (mg/dL)**	**p-value**
Short Sleep (<6 hours)	210.5 ± 24.3	
Optimal Sleep (7-8 hours)	190.8 ± 18.7	0.008
Long Sleep (>9 hours)	205.7 ± 22.6	

**Table 10:** Time to fall asleep and cardiovascular risk

**Time to Fall Asleep**	**Framingham Risk Score (Mean ± SD)**	**p-value**
<15 minutes	11.2 ± 2.8	
15-30 minutes	12.5 ± 3.2	0.041
>30 minutes	14.1 ± 3.5	
